# Reply to: Exegesis on surgery for adrenal gland diseases

**DOI:** 10.1016/j.sopen.2026.10.001

**Published:** 2026-10-09

**Authors:** Maria Irene Bellini, Immacolata Iannone, Gaia Cicioni, Daniele Crocetti, Paolo Sapienza

**Affiliations:** Department of Surgery, Sapienza University of Rome, Italy

We read with interest the correspondence by Sengul and Sengul [1].

We agree that robotic surgery can be justified when its added immediate expense is matched by a clinical or technical advantage, rather than used routinely for cases that laparoscopy already handles well. For example, in the elective cholecystectomy, the robotic platform could be seen as cost-intensive, with longer operative times and only a clinically negligible difference in mean blood loss compared with laparoscopy. On that basis, the economic impact requires justification through improved outcomes or greater case complexity [2].

In our study [3], we describe the 16-year evolution of adrenal surgery at a tertiary referral center, without the aim to establish the superiority of robotic over laparoscopic adrenalectomy (LA). The observed temporal evolution did not represent a progressive replacement of open surgery by robotics. Between 2009 and 2020, LA accounted for 87% of procedures, robotic adrenalectomy (RA) for 3%, and open adrenalectomy for 10%. During 2021–2025, RA accounted for 63%, LA for 24%, and open surgery for 13% [3]. Thus, the proportion of open procedures remained relatively stable, whereas a substantial redistribution occurred within minimally invasive surgery (MIS). These findings should therefore be interpreted as an intra-MIS technological transition rather than an expansion of minimally invasive indications.

Regarding perioperative outcomes, we agree that our data do not demonstrate a clinically meaningful advantage of RA over LA in terms of blood loss. Mean blood loss was 62 ± 20 mL following RA and 66 ± 28 mL following LA, whereas operative time was significantly shorter with laparoscopy (82 ± 26 vs 124 ± 26 min) [3]. The difference in blood loss between the two MIS approaches did not reach statistical significance [3].

Nevertheless, these findings should be interpreted in the context of the different maturity of the two programs. LA represents a long-established technique at our institution, whereas RA was introduced more recently. At the time of analysis, our robotic experience comprised 27 procedures and was still within the implementation phase of the program [3]. Therefore, our findings should not be considered evidence of robotic superiority, but neither do they allow a definitive comparison between a mature laparoscopic program and a recently implemented robotic program.

Current evidence supports this cautious interpretation. A recent meta-analysis comparing RA and LA found broadly comparable perioperative outcomes, with modest advantages for RA in blood loss, conversion rate, and length of stay, but at higher hospitalization costs [4]. Therefore, RA should be regarded as a complementary rather than universally superior minimally invasive approach.

The authors also raise an important concern regarding oncological safety and the use of MIS for large adrenal lesions. We strongly agree that technological capability should never supersede oncological principles. However, tumor size, suspicion of malignancy, and evidence of local invasion are related but not interchangeable criteria.

Only two lesions larger than 120 mm, representing 0.7% of our entire cohort, were treated using MIS, whereas lesions ≥100 mm were predominantly managed through an open approach [3]. Furthermore, more than half of the tumors approached by laparotomy were incidentalomas, mainly because of their larger size and suspicion of malignancy [3]. Therefore, these two selected cases should not be interpreted as evidence of a systematic extension of MIS indications or of “technology creep.”

Current recommendations similarly emphasize that oncological characteristics, rather than technological feasibility alone, should determine the surgical approach. Open adrenalectomy remains the standard approach for confirmed or highly suspected adrenocortical carcinoma, particularly in the presence of local invasion, whereas MIS should be considered only in appropriately selected cases and experienced centers [5], [6].

Importantly, open adrenalectomy maintained a stable role throughout our study period and remained deliberately selected for large tumors, suspected malignancy, and locally advanced disease [3]. This supports the concept that the introduction of robotics at our institution did not result in an indiscriminate expansion of MIS indications, but rather modified the choice of technique within an already established minimally invasive program.

We also appreciate the proposed “dual-gatekeeper” framework [1] and agree with its underlying principle that access to advanced technology should not itself constitute an indication for its use. However, we believe that robotic surgery should be considered selectively whenever its technical characteristics may offer a meaningful advantage in challenging cases, while preserving patient safety and oncological principles.

Ultimately, open, laparoscopic, and robotic adrenalectomy should not be considered competing strategies, but rather complementary components of the contemporary adrenal surgeon's armamentarium [7]. Conventional laparoscopy remains a mature and effective minimally invasive approach; robotics represents an additional platform whose role continues to evolve; and open adrenalectomy remains indispensable whenever dictated by tumor biology, local invasion, anatomical complexity, or oncological principles.

In summary, we support surgical innovation driven by therapeutic necessity rather than technological momentum. The value of robotic adrenalectomy lies not in replacing established laparoscopic or open surgery, but in its selective integration into a patient-centered surgical strategy based on case complexity, institutional expertise, economic sustainability, and oncological safety.

## CRediT authorship contribution statement

**Maria Irene Bellini:** Conceptualization, Data curation, Writing – original draft. **Immacolata Iannone:** Conceptualization, Data curation, Writing – original draft. **Gaia Cicioni:** Writing – review & editing. **Daniele Crocetti:** Writing – review & editing. **Paolo Sapienza:** Conceptualization, Writing – review & editing.

## Declaration of competing interest

The authors declare no conflict of interest.
